# Supplementary material for: Admixture of evolutionary rates across a butterfly hybrid zone
Source: eLife. 2022 Jun 15;11:e78135. doi: 10.7554/eLife.78135 (PMC9246367; doi:10.7554/eLife.78135)
Supplement: Transparent reporting form [file elife-78135-transrepform1.docx]

***eLife’s* transparent reporting form**

We encourage authors to provide detailed information *within their submission* to facilitate the interpretation and replication of experiments. Authors can upload supporting documentation to indicate the use of appropriate reporting guidelines for health-related research (see [EQUATOR Network](http://www.equator-network.org/%20)), life science research (see the [BioSharing Information Resource](https://biosharing.org/)), or the [ARRIVE guidelines](http://www.plosbiology.org/article/info:doi/10.1371/journal.pbio.1000412) for reporting work involving animal research. Where applicable, authors should refer to any relevant reporting standards documents in this form.

If you have any questions, please consult our Journal Policies and/or contact us: [editorial@elifesciences.org](mailto:editorial@elifesciences.org).

**Sample-size estimation**

- You should state whether an appropriate sample size was computed when the study was being designed
- You should state the statistical method of sample size computation and any required assumptions
- If no explicit power analysis was used, you should describe how you decided what sample (replicate) size (number) to use

Please outline where this information can be found within the submission (e.g., sections or figure legends), or explain why this information doesn’t apply to your submission:

Approximately 4 diploid individuals per population is the original design in sampling for whole-genome resequencing. This size is suitable for window-based population genetic statistics (Fst, Dxy, Pi, local ancestry, entropy) and phylogenetic trees, but not sufficient for site-specific statistics (allele frequencies, linkage disequilibrium)

In reality, as these butterflies are not abundant, it is practically impossible to collect more individuals than those used in the study during the designated field trip

Sample size from each population is shown in Figure 2B.

**Replicates**

- You should report how often each experiment was performed
- You should include a definition of biological versus technical replication
- The data obtained should be provided and sufficient information should be provided to indicate the number of independent biological and/or technical replicates
- If you encountered any outliers, you should describe how these were handled
- Criteria for exclusion/inclusion of data should be clearly stated
- High-throughput sequence data should be uploaded before submission, with a private link for reviewers provided (these are available from both GEO and ArrayExpress)

Please outline where this information can be found within the submission (e.g., sections or figure legends), or explain why this information doesn’t apply to your submission:

**Replication 1:** local ancestry estimation (technical replication)

Number of replicates: 50 times per chromosome

Software: ELAI

Detail: For each chromosome, we repeatedly estimate its local ancestry for individuals from population WN and BJ using parental panels (populations XY and KM). For each estimated local ancestry signal, we compute its entropy and used all replicated estimates simultaneously in calculating the correlation coefficients (Figure 3E). In this way, we control for the stochasticity in ancestry estimation and will not inflate correlation coefficients.

**Replication 2:** Entropy simulation on ideal chromosomes (technical replication)

Number of replicates: 1000

Software: custom scripts using Julia

Detail: Idealized chromosomes with 1000 equally spaced SNPs. Different chromosomes are unrelated. Each parameter combination (parental contributions & ancestry disassociation probability between adjacent SNPs) is simulated for 1000 times.

**Replication 3:** Bootstrap support of local gene trees (technical replication)

Number of replicates: 5000 (ultrafast bootstrap)

Software: IQTREE - 2.0

Detail: For each local 50kb genomic window, IQTREE estimates the support for each topology using ultrafast bootstrap. (Mentioned in Materials and Methods)

**Replication 4:** Simulated models of rate-mixing (technical replication)

Number of replicates: 10^6 for two-population models, 10^4 for stepping stone models

Software: custom scripts using Julia

Detail: Summarized in Figure 6 - Supplements 1-3

**Statistical reporting**

- Statistical analysis methods should be described and justified
- Raw data should be presented in figures whenever informative to do so (typically when N per group is less than 10)
- For each experiment, you should identify the statistical tests used, exact values of N, definitions of center, methods of multiple test correction, and dispersion and precision measures (e.g., mean, median, SD, SEM, confidence intervals; and, for the major substantive results, a measure of effect size (e.g., Pearson's r, Cohen's d)
- Report exact p-values wherever possible alongside the summary statistics and 95% confidence intervals. These should be reported for all key questions and not only when the p-value is less than 0.05.

Please outline where this information can be found within the submission (e.g., sections or figure legends), or explain why this information doesn’t apply to your submission:

Figure 2C: Node supports for the mitochondrial tree is displayed in the figure

Figure 3A: Raw data, estimated density, mean, and standard errors are simultaneously displayed for Dxy and pi

Figure 3C: Mean, and upper/lower deviations from the mean are explained in legend

Figure 3E: Statistical test using jackknifing is explained in the legend

Figure 4: For clarity, only mean and standard error are displayed. Z-scores can be found in the supplementary figure and source data

Figure 5C: The test (Wilcoxon signed rank test) is explained in Supplementary File 2 along with with test statistics and p-values

Figure 6D: Goodness-of-fit (R^2) is displayed in figures, along with estimated r0 and its standard error.

(For large datasets, or papers with a very large number of statistical tests, you may upload a single table file with tests, Ns, etc., with reference to sections in the manuscript.)

**Group allocation**

- Indicate how samples were allocated into experimental groups (in the case of clinical studies, please specify allocation to treatment method); if randomization was used, please also state if restricted randomization was applied
- Indicate if masking was used during group allocation, data collection and/or data analysis

Please outline where this information can be found within the submission (e.g., sections or figure legends), or explain why this information doesn’t apply to your submission:

Samples were allocated based on their geographic locality (Figure 2B)

**Additional data files (“source data”)**

- We encourage you to upload relevant additional data files, such as numerical data that are represented as a graph in a figure, or as a summary table
- Where provided, these should be in the most useful format, and they can be uploaded as “Source data” files linked to a main figure or table
- Include model definition files including the full list of parameters used
- Include code used for data analysis (e.g., R, MatLab)
- Avoid stating that data files are “available upon request”

Please indicate the figures or tables for which source data files have been provided:

Figure 2, Figure 3, Figure 4, Figure 5 contain individually uploaded source data files.

Figure 6 does not have such source data file as raw numbers are already displayed in the figure.
